# Supplementary material for: In-Scanner Thoughts shape Resting-state Functional Connectivity: how participants “rest” matters
Source: bioRxiv. 2024 Jun 6:2024.06.05.596482. Preprint. [Version 1] doi: 10.1101/2024.06.05.596482 (PMC11188111; doi:10.1101/2024.06.05.596482)
Supplement: Supplement 1 [file media-1.pdf]

Supplementary Tables

Year	Title	Clinical Condition	Healthy Controls (N)	Patients (N)	B0	Dur(s)	TR(s)	Edge Threshold	Size	# Permut.	Network Threshold	# Nodes	# Edges	# Sig. Edges	% Sig. Edges	# Sig. Nodes	% Sig. Nodes
2010	Network-based statistic: Identifying differences in brain networks	Schizophrenia	15	12	1.5T	1024	2	T > 3.0	Extent	5000	pFWE = 0.037	74	2701	40	0.015	29	0.3919
2013	Decreased Functional Brain Connectivity in Adolescents with Internet Addiction	Internet Addiction	11	12	3.0T	405	2.7	T > 3.0	Extent	20000	pFWE < 0.05	90	4005	59	0.015	38	0.422
2013	Disrupted topological organization in whole-brain functional networks of heroin-dependent individuals: A resting-state fMRI study	Heroin Addiction	15	17	1.5T	480	2	T > 1.7	Extent	1000	pFWE < 0.001	90	4005	19	0.005	19	0.211
2014	Altered brain network modules induce helplessness in major depressive disorder	Major Depressive Disorder	16	16	3.0T	300	3		Extent	5000	pFWE < 0.05	90	4005	24	0.006	24	0.267
2014	Disruption of structure-function coupling in the schizophrenia connectome	Schizophrenia	17	17	3.0T	600	2.4	T > 3.0	Extent	10000	pFWE = 0.011	90	4005	86	0.021	72	0.8
2015	Connectome-scale assessments of functional connectivity in children with primary monosymptomatic nocturnal enuresis	Nocturnal Enuresis	29	24	3.0T	420	2	T > 1.6	-	10000	pFWE < 0.05	90	4005	10	0.002	7	0.078
2015	Disrupted brain network topology in pediatric posttraumatic stress disorder: A resting-state fMRI study	Posttraumatic Stress Disorder	24	24	3.0T	400	2	T > 2.0	-	10000	pFWE = 0.007	90	4005	7	0.002	13	0.144
2015	Frequency dependent topological alterations of intrinsic functional connectome in major depressive disorder	Major Depressive Disorder	50	50	3.0T	484	2	p <0.001	Extent	10000	pFWE < 0.05	112	6216	23	0.004	13	0.116
2015	Network topology and functional connectivity disturbances precede the onset of Huntington's disease	Huntington's Disease (medium disease burden)	16	16	3.0T	369.6	2.8	T > 3.5	-	5000	pFWE < 0.05	300	44850	96	0.002	-	-
		Huntington's disease (high disease burden)												252	0.006	-	-
2015	Reduced functional connectivity in the thalamo-insular subnetwork in patients with acute anorexia nervosa	Anorexia Nervosa	35	35	3.0T	418	2.2	T > 4.0	Extent	5000	pFWE < 0.05	104	5356	7	0.001	7	0.067
2016	Aberrant functional brain connectome in people with antisocial personality disorder	Antisocial Personality Disorder	32	32	1.5T	300	2	p < 0.0005	Extent	10000	pFWE = 0.001	90	4005	47	0.012	38	0.422
2016	Altered functional connectivity of the default mode network in Williams syndrome: a multimodal approach	Williams Syndrome	7	7	3.0T	300	3	p <0.01	Extent	5000	pFWE = 0.027	82	3321	22	0.007	21	0.256

2016	Brain Connectomics' Modification to Clarify Motor and Nonmotor Features of Myotonic Dystrophy Type 1	Myotonic Dystrophy Type 1	26	31	3.0T	440	2.08		-	50000	pFWE < 0.005	116	6670	83	0.012	51	0.44
2016	Disrupted topological organization of structural and functional brain connectomes in clinically isolated syndrome and multiple sclerosis	Multiple Sclerosis	35	41	3.0T	360	2	p < 0.05	Extent	10000	pFWE < 0.05	90	4005	38	0	22	0
2016	Distinct disruptions of resting-state functional brain networks in familial and sporadic schizophrenia	Schizophrenia (familiar)	26	26	3.0T	370	2	T > 2.01	-	10000	pFWE = 0.022	90	4005	13	0.003	13	0.144
		Schizophrenia (sporadic)									pFWE = 0.033			12	0.003	12	0.133
2016	Large-scale hypoconnectivity between resting-state functional networks in unmedicated adolescent major depressive disorder	Major Depressive Disorder	56	55	3.0T	512	2	T > 1.659, p < 0.05	Extent	100000	pFWE=0.0237	17	136	7	0.051	8	0.471
2016	Network analysis of functional brain connectivity in borderline personality disorder using resting-state fMRI	Borderline Personality Disorder	10	20	3.0T	360	2	T > 3.5	Extent	10000	pFWE = 0.0304	82	3321	26	0.017	26	0.488
2016	Resting state brain network disturbances related to hypomania and depression in medication-free bipolar disorder	Bipolar Disorder	30	60	3.0T	333	2.25	T > 3.35	Extent	5000	pFWE < 0.05	181	16290	61	0.004	48	0.2652
2016	Whole-brain analytic measures of network communication reveal increased structure-function correlation in right temporal lobe epilepsy	Epilepsy	13	7	3.0T	1200	3.6	T > 5.0	Extent	10000	pFWE < 0.05	512	130816	104	0.001	38	0.074
2017	Altered functional network architecture in orbitofronto-striato-thalamic circuit of unmedicated patients with obsessive-compulsive disorder	Obsessive Compulsive Disorder	61	61	3.0T	413	3.5	p < 0.05	-	10000	pFWE < 0.05	17	136	8	0.059	10	0.5882
2017	Altered intrinsic functional brain architecture in female patients with bulimia nervosa	Bulimia Nervosa	45	48	3.0T	420	2		-	10000	-	90	4005	103	0.026	21	0.2333
2017	Altered network efficiency of functional brain networks in patients with breast cancer after chemotherapy	Breast Cancer	40	28	-	-	-	p < 001	Extent	10000	-	90	4005	79	0.02	35	0.3889
2017	Cognitive phenotypes in parkinson's disease differ in terms of brain-network organization and connectivity	Parkinson's Disease (G1 > G2)	31	32	3.0T	600	2.4	p < 0.005	Extent	20000	-	164	13366	19	0.001	20	0.122
		Parkinson's Disease (G1 > G3)		43										63	0.005	63	0.3841
2017	Cognitive phenotypes in parkinson's disease differ in terms of brain-network organization and connectivity	Parkinson's Disease (G1 > G4)	31	14	3.0T	600	2.4	p < 0.005	Extent	20000	-	164	13366	172	0.013	128	0.7805
2017	Decreased functional connectivity within a language subnetwork in benign epilepsy with centrotemporal spikes	Epilepsy	20	25	3.0T	368	2	T > 3.0	Intensity	50000	-	90	4005	41	0.01	36	0.4
2017	Discriminating cognitive status in Parkinson's disease through functional connectomics and machine learning	Parkinson's Disease	38	27	3.0T	600	2	-	Extent	10000	-	246	30135	235	0.008	120	0.4878

2017	Disrupted functional connectome in antisocial personality disorder	Antisocial Personality Disorder	32	32	1.5T	300	2	p < 5e-4	Extent		-	90	4005	47	0.012	38	0.4222
2017	Disrupted resting-state brain network properties in obesity: Decreased global and putaminal cortico-striatal network efficiency	Obesity	40	40	3.0T			T > 3.0	Extent	10000	-	90	4005	30	0.007	19	0.2111
2017	Evaluation of Whole-Brain Resting-State Functional Connectivity in Spinal Cord Injury: A Large-Scale Network Analysis Using Network-Based Statistic	Spinal Cord Injury	15	15	3.0T	480	2	-	-	-	-	116	6670	21	0.003	20	0.1724
2017	Neural substrates of motor and cognitive dysfunctions in SCA2 patients: A network based statistics analysis	Spinocerebellar Ataxia Type 2	33	9	3.0T	440	2.08	-	-	5000	pFWE < 0.05	116	6670	110	0.016	62	0.5345
2017	Reduced orbitofrontal-thalamic functional connectivity related to suicidal ideation in patients with major depressive disorder	Major Depressive Disorder	36	36	3.0T	360	3	T > 3.0	Extent	5000	pFWE < 0.05	90	4005	6	0.001	7	0.0778
2017	Structural and functional hyperconnectivity within the sensorimotor system in xenomelia	Xenomelia	13	13	3.0T	600	4	T > 3.6	Extent	5000	-	116	6670	24	0.004	27	0.2328
2017	Tackling variability: A multicenter study to provide a gold-standard network approach for frontotemporal dementia	Fronto-Temporal Dementia (Country 1)	16	16	1.5T	600	2.7	T > 3.0	Extent	5000	-	90	4005	51	0.013	42	0.4667
		Fronto-Temporal Dementia (Country 2)	29	17	3.0T	300	3	T > 3.0						83	0.021	56	0.6222
		Fronto-Temporal Dementia (Country 3)	15	12	3.0T	420	2	T > 4.0						40	0.01	35	0.3889
2017	Topologically convergent and divergent functional connectivity patterns in unmedicated unipolar depression and bipolar disorder	Unipolar Depression	31	43	-	-	-	p < 0.001	Extent	5000	-	1024	523776	51	0	44	0.043
		Bipolar Disorder	32											81	0	74	0.0723
2018	Altered brain functional connectome in migraine with and without restless legs syndrome: A resting-state functional MRI study	Migraine w/o RLS	19	22	3.0T	500	2.5	p < 0.005	Extent	5000	pFWE < 0.05	236	27730	162	0.006	121	0.5127
		Migraine with RLS												136	0.005	82	0.3475
2018	Altered cerebellar-insular-parietal-cingular subnetwork in adolescents in the earliest stages of anorexia nervosa: A network-based statistic analysis	Anorexia Nervosa	15	15	1.5T	284.8	3.56	T > 3.1	-	10000	pFWE < 0.05	128	8128	31	0.004	30	0.2344
2018	Brain-behavior patterns define a dimensional biotype in medication-naïve adults with attention-deficit hyperactivity disorder	Attention Deficit Hyperactivity Disorder	123	80	3.0T	459	2.55	T > 3.5	-	10000	pFWE < 0.05	253	31878	41	0.001	40	0.1581
2018	Evaluating functional connectivity alterations in autism spectrum disorder using network-based statistics	Autism Spectrum Disorder	82	74	3.0T	360	2	T > 3.0	Extent	5000	pFWE < 0.025	116	6670	158	0.024	105	0.9052

2018	Impact of Zika Virus on adult human brain structure and functional organization	Zika Virus	9	9	3.0T	360	2	T > 3.6	Extent	5000	-	160	12720	19	0.001	20	0.125
2018	Progressively disrupted brain functional connectivity network in subcortical ischemic vascular cognitive impairment patients	Subcortical Ischemic Vascular Cognitive Impairment w/o Dementia	19	20	3.0T	480	2	p < 0.005	Extent	10000	-	90	4005	93	0.023	38	0.4222
		Subcortical Ischemic Vascular Cognitive Impairment with Dementia												141	0.035	66	0.7333
2018	Short- and long-range synergism disorders in lifelong premature ejaculation evaluated using the functional connectivity density and network property	Alcohol Dependent	36	21	3.0T	360	3	T > 3.0	Extent	10000	pFWE < 0.05	90	4005	373	0.093	69	0.7667
2019	Aberrant brain network topology in fronto-limbic circuitry differentiates euthymic bipolar disorder from recurrent major depressive disorder	Bipolar Disorder	30	20	3.0T	600	2	T > 3.4	Intensity	10000	pFWE < 0.05	90	4005	11	0.003	11	0.1222
			42	57		480		T > 3.1	Extent					85	0.021	68	0.7556
2019	Brain functional connectivity is altered in patients with Takotsubo Syndrome	Takotsubo Syndrome	8	8	1.5T	380	2	T > 4.14		5000	pFWE < 0.05	268	35778	39	0.001	36	0.1343
2019	Cognitive performance in mid-stage Parkinson’s disease: functional connectivity under chronic antiparkinson treatment	Parkinson's Disease	16	16	3.0T		2	T > 6.0	Intensity	5000	pFWE < 0.05	206	21115	15	0.001	20	0.0971
2019	Data-Driven Clustering Reveals a Link Between Symptoms and Functional Brain Connectivity in Depression	Depression	72	178	3.0T	500	2.5	p < 0.05		10000	pFWE < 0.05	19	171	22	0.129	15	0.7895
2019	Disrupted topological organization of human brain connectome in diabetic retinopathy patients	Diabetic Retinopathy	38	35	3.0T		2	T > 2.649	-	10000	-	90	4005	52	0.013	40	0.4444
2019	Early functional connectivity predicts recovery from visual field defects after stroke	Stroke	15	32	3.0T	240	2	T > 3.0	Extent	5000	pFWE < 0.05	82	3321	9	0.003	6	0.0732
2019	Functional brain connectome and its relation to mild cognitive impairment in cerebral small vessel disease patients with thalamus lacunes: A cross-sectional study	Cerebral Small Vessel Disease with Thalamus Lacunes	34	14	3.0T	420	2	T > 2.02	-	10000	-	90	4005	13	0.003	6	0.0667
2019	Functional network-based statistics reveal abnormal resting-state functional connectivity in minimal hepatic encephalopathy	Anorexia Nervosa	19	19	3.0T	360	2	T > 3.1	-	5000	pFWE < 0.05	90	4005	43	0.011	29	0.3222
2019	Resting-state functional connectivity after concussion is associated with clinical recovery	Concussion	60	62	3.0T	360.72	0.72	T > 2.5	-	10000	pFWE < 0.05	200	19900	1464	0.074	180	0.9

Supplementary Table 1 | Studies selected for literature review of prior NBS-based clinical studies in human populations.

Supplementary Table 2.

Year	Title	Category	Prediction Target	# Subjects	Atlas	# ROIs	Model Accuracy (r)						Cross Validation
							Positive Network		Negative Network		Pos + Neg Networks		
							Pearson	Spearman	Pearson	Spearman	Pearson	Spearman	
2018	Individualized prediction of trait narcissism from whole-brain resting-state functional connectivity	Personality	Narcissism	168	Shen 268-node	268	**0.24**	-	-	-	-	-	LOOCV
2018	Resting-state functional connectivity predicts cognitive impairment related to Alzheimer's disease	Clinical	Alzheimer's Disease Assessment Scale (ADAS 11) Score	59	Shen 268-node	268	-	**0.49**	-	0.27	-	-	LOOCV
2018	Resting-state functional connectivity predicts neuroticism and extraversion in novel individuals	Personality	Neuroticism	114	Shen 268-node	244	**0.27**	-	0.14	-	**0.22**	-	LOOCV
			Extraversion				**0.22**	-	0.2	-	**0.22**	-	
2019	Connectome-based individualized prediction of loneliness	Cognitive	Loneliness	75	Shen 268-node	268	-0.3	-	**0.244**	-	-	-	LOOCV
2019	The Functional Brain Organization of an Individual Allows Prediction of Measures of Social Abilities Transdiagnostically in Autism and Attention-Deficit/Hyperactivity Disorder	Cognition	Processing Speed	99	Shen 268-node	268	-	**0.35**	-	**0.4**	-	**0.38**	10-Fold CV
		Clinical	Social Responsiveness Scale (SRS) Total Score				-	-	-	-	**0.32**	-	LOOCV
			SRS Communication score				-	-	-	-	**0.3**	-	
			SRS Motivation score				-	-	-	-	**0.23**	-	
			SRS Mannerisms score				-	-	-	-	**0.37**	-	
			SRS Awareness score				-	-	-	-	**0.27**	-	
			SRS Cognition score				-	-	-	-	**0.27**	-	
			Autism Diagnostic Observation Scale (ADOS) Total score				-	-	-	-	**0.43**	-	
			Ados Social Affect Score				-	-	-	-	**0.53**	-	
			Ados Generic Total Score				-	-	-	-	**0.4**	-	
			Ados Severity Score				-	-	-	-	**0.6**	-	
2020	Behavioral and brain signatures of substance use vulnerability in childhood	Clinical	Substance Risk Seeking	3193	Shen 268-node	268	-	-	-	-	**0.1**	-	10-Fold CV
			Familial Risk For Substance Abuse	3193			-	-	-	-	-	-	

2020	Brain functional connectome-based prediction of individual decision impulsivity	Personality	Decision Impulsivity (Ddis_AUC_40K)	809	Group-ICA	200	**0.25**	-	**0.24**	-	-	-	LOOCV
			Decision Impulsivity (Ddis_AUC_200)				**0.23**	-	0.19	-	-	-	
2020	Connectome-based models can predict early symptom improvement in major depressive disorder	Clinical	Depression Severity	108	Shen 268-node	268	-	-	**0.41**	-	-	-	LOOCV
2020	Connectome-based models can predict processing speed in older adults	Cognitive	Processing Speed	99	Shen 268-node	268	-	**0.36**	-	**0.42**	-	**0.4**	LOOCV
2020	Do intrinsic brain functional networks predict working memory from childhood to adulthood?	Cognitive	Working Memory	87 - Preschoolers	Shen 268-node	268	-	-	-	-	-	0.118	5-Fold CV
				117 - Early school age			-	-	-	-	-	0.04	
				110 - Late school age			-	-	-	-	-	-0.009	
				95 - Adolescents			-	-	-	-	-	**0.228**	
				59 - Adults			-	-	-	-	-	**0.312**	
2020	Individualized Prediction of PTSD Symptom Severity in Trauma Survivors From Whole-Brain Resting-State Functional Connectivity	Clinical	PTSD Symptoms	64	AAL	268	-	**0.3**	-	**0.17**	-	-	LOOCV
2020	Multimodal data revealed different neurobiological correlates of intelligence between males and females	Cognitive	Intelligence Quotient	166 - Males	AAL	116	**0.22**	-	**0.27**	-	**0.28**	-	LOOCV
				160 - Females			**0.23**	-	**0.33**	-	**0.36**	-	
2020	Predicting Patient Reported Outcomes of Cognitive Function Using Connectome-Based Predictive Modeling in Breast Cancer	Clinical	Executive Dysfunction	76	Shen 268-node	268	-	-	-	-	**0.65**	-	10-Fold CV
			Memory Functions				-	-	-	-	**0.32**	-	
2020	Preliminary prediction of individual response to electroconvulsive therapy using whole-brain functional magnetic resonance imaging data	Clinical	Change In Depression Scores	122	Brainnetome Atlas	246	-	**0.27**	-	**0.51**	-	**0.46**	LOOCV
2020	Robust prediction of individual personality from brain functional connectome	Personality	Agreeableness	1003	ICA derived nodes	200	**0.163**	-	0.115	-	-	-	LOOCV
			Openness				-0.135	-	0.138	-	-	-	
			Conscientiousness				0.124	-	**0.281**	-	-	-	
			Neuroticism				0.097	-	-0.085	-	-	-	
			Extraversion				-0.037	-	-0.078	-	-	-	
202		Cognitive	Digit Span Forward	91	AAL	90	0.083	-	**0.51**	-	**0.25**	-	LOOCV

	The individualized prediction of cognitive test scores in mild cognitive impairment using structural and functional connectivity features		Digit Span Backward				**0.49**	-	**0.6**	-	**0.59**	-	
			Immediate Recall				**0.38**	-	**0.54**	-	**0.51**	-	
			Delayed Recall				**0.35**	-	**0.46**	-	**0.43**	-	
			Recognition				**0.33**	-	0.099	-	**0.31**	-	
			Color Trails Part 1				**0.55**	-	**0.58**	-	**0.56**	-	
			Color Trails Part 2				**0.6**	-	**0.65**	-	**0.63**	-	
			Color Trains Test Inference				0.11	-	**0.41**	-	0.21	-	
			Wais-Iii Block Design				**0.55**	-	**0.45**	-	**0.48**	-	
			Mini-Mental State Examination				0.51	-	**0.52**	-	**0.54**	-	
2021	Connectome-based model predicts episodic memory performance in individuals with subjective cognitive decline and amnestic mild cognitive impairment	Cognition	Auditory/Verbal Test Delayed Call	83	AAL	116	0.033	-	**0.22**	-	-	-	LOOCV
2021	Connectome-based prediction of global cognitive performance in people with HIV	Clinical	Global Cognitive Measure	67	Shen 268-node	268	**0.35**	-	**0.382**	-	**0.464**	-	LOOCV
2021	Connectome-Based Predictive Modeling of Creativity Anxiety	Clinical	Creativity Anxiety	281	Shen 268-node	268	**0.18**	-	**0.2**	-	**0.19**	-	LOOCV
2021	Connectome-Based Predictive Modeling of Individual Anxiety	Clinical	Anxiety	76	Shen 268-node	268	-	-0.01	-	**0.33**	-	-	LOOCV
2021	The functional and structural connectomes associated with geriatric depression and anxiety symptoms in mild cognitive impairment: Cross-syndrome overlap and generalization	Clinical	Geriatric Depression Scale (GDS)	91	Brainnetome atlas	246	-	**0.331**	-	**0.314**	-	**0.357**	LOOCV
			Geriatric Anxiety Inventory (GAI)				-	**0.198**	-	**0.308**	-	**0.308**	
2021	The functional connectome predicts feeling of stress on regular days and during the COVID-19 pandemic		Perceived Stress	673	Brainnetome Atlas	246	**0.36**	-	**0.41**	-	**0.44**	-	LOOCV
2022	Connectome-based model predicts individual psychopathic traits in college students	Personality	T-PTS (Psychopathic traits)	84	Dosenbach	142	-	0.073	-	**0.295**	-	**0.308**	LOOCV and k fold
			S-PTS (Psychopathic traits)				-	0.129	-	**0.309**	-	**0.353**	
			P-PTS (Psychopathic traits)				-	0.008	-	0.07	-	0.099	
2022	Connectome-based prediction of marital quality in husbands' processing of spousal interactions	Well-being	Marital Quality	25 - Males	Shen 268-node	268	-0.15	-	-0.08	-	-0.31	-	LOOCV
				25 - Females			-0.39	-	0.17	-	-0.07	-	

2022	Connectome-based predictive models using resting-state fMRI for studying brain aging	Demographic	Age	256	Shen 268-node	268	**0.398**	-	**0.505**	-	**0.484**	-	LOOCV
2022	Individualized prediction of consummatory anhedonia from functional connectome in major depressive disorder	Clinical	Anhedonia	54	Shen 268-node	268	**0.28**	-	-	-	-	-	10-Fold CV
2022	Multi-modality connectome-based predictive modeling of individualized compulsions in obsessive-compulsive disorder	Clinical	Compulsion	54	Shen 268-node	268	-	-	**0.33**	-	-	-	10-Fold CV
			YBOCS Total Score				-	-	**0.38**	-	**0.41**	-	LOOCV
2022	Neural mechanisms underlying empathy during alcohol abstinence: evidence from connectome-based predictive modeling	Personality	Empathy	59	Dosenbach	160	-	-	-	-	**0.24**	-	LOOCV
2022	Predicting children's math skills from task-based and resting-state functional brain connectivity	Cognition	Math Skills	31	Brainnetome Atlas	210	-	-	-	-	**0.54**	-	LOOCV
2022	Preoperative brain connectome predicts postoperative changes in processing speed in moyamoya disease	Clinical	Post-Operative Processing Speed (1 Month)	12	AAL	116	-	**0.63**	-	0.31	-	-	LOOCV
			Post-Operative Processing Speed (6 Month)				-	**0.62**	-	**0.55**	-	-	
2023	A connectome-based neuromarker of nonverbal number acuity and arithmetic skills	Cognitive	Numeracy	154	Shen 268-node	268	**0.24**	-	0.032	-	-	-	LOOCV
2023	Age-related intrinsic functional connectivity underlying emotion utilization	Personality	Emotion Utilization	133	Shen 268-node	219	-	**0.29**	-	**0.2**	-	-	LOOCV
2023	Brain connectivity in frailty: Insights from The Irish Longitudinal Study on Ageing (TILDA)		Frailty	347	Shen 268-node	268	-	**0.169**	-	**0.195**	-	-	10-Fold CV
2023	Brief intensive social gaze training reorganizes functional brain connectivity in boys with fragile X syndrome	Clinical	FXS vs. ASD	37	Corgon Atlas	333	-	**0.37**	-	**0.44**	-	-	LOOCV
2023	Connectome-based fingerprint of motor impairment is stable along the course of Parkinson's disease	Clinical	Levels Of Motor Impairment	81	Shen 268-node	268	-	**0.21**	-	0.16	-	-	LOOCV
2023	Connectome-based modeling reveals a resting-state functional network that mediates the relationship between social rejection and rumination	Cognitive	Rumination	560	400 ROI Schaefer	400	0.045	-	**0.152**	-	-	-	LOOCV

2023	Connectome-based prediction of eating disorder-associated symptomatology	Clinical	Body Image Concerns	660	Shen 268-node	268	-	**0.11**	-	**0.11**	-	**0.14**	10-Fold CV
			Binge Eating Frequency				-	0.06	-	0.01	-	0.04	
			Compensatory Behaviors				-	0.07	-	**0.09**	-	**0.1**	
2023	Connectome-based prediction of the severity of autism spectrum disorder	Clinical	Communication	359 - 323 ASD, 36 controls	Dosenbach Atlas	160	-	-0.18	-	**0.22**	-	-	LOOCV
			Social Interaction	359 - 323 ASD, 36 controls			-	**0.21**	-	0.12	-	-	
			Stereotyped Behavior	359 - 323 ASD, 36 controls			-	-0.02	-	0.12	-	-	
2023	Connectome-based predictive modeling of compulsion in obsessive-compulsive disorder	Clinical	Compulsion	57	Shen 268-node	268	-	**0.3**	-	**0.32**	-	0.3	LOOCV
2023	Connectome-based predictive modeling of fluid intelligence: evidence for a global system of functionally integrated brain networks	Cognitive	Fluid Intelligence	159	Glasser Atlas	-	**0.42**	-	-	-	-	-	LOOCV
2023	Connectome-based predictive modeling of trait forgiveness	Personality	Trait Forgiveness	100	Shen 268-node	268	**0.23**	-	**0.25**	-	**0.31**	-	10-Fold CV
2023	Connectome-based predictive modeling predicts paranoid ideation in young men with paranoid personality disorder: a resting-state functional magnetic resonance imaging study	Clinical	Paranoia	18	Brainnetome Atlas	246	-	−0.0926	-	**0.684**	-	0.3148	LOOCV
2023	Functional brain connectivity predicts sleep duration in youth and adults	Cognitive	Sleep Duration	652 - HCP	Shen 268-node	268	-	-	-	-	-	**0.164**	10-Fold CV
				786 - ABCD			-	-	-	-	-	**0.238**	
2023	Functional connectome predicting individual gait function and its relationship with molecular architecture in Parkinson's disease	Clinical	Gait Function	48	Shen 268-node	268	-	-	-	-	**0.31**	-	LOOCV
2023	Functional connectomes of akinetic-rigid and tremor within drug-naïve Parkinson's disease	Clinical	AR	81	Shen 268-node	268	-	-	-	-	-	**0.28**	LOOCV
			Tremor				-	-	-	-	-	**0.32**	
2023	Neural correlates of schizotypal traits: Findings from connectome-based predictive modelling	Clinical	Schizophrenia Traits	82	Shen 268-node	268	-0.23	-	**0.29**	-	-	-	LOOCV
2023	Task and Resting-State Functional Connectivity Predict Driving Violations	Personality	Sensation Seeking	29	Shen 268-node	178	0.05	-	-0.03	-	-	-	LOOCV
			Impulsivity				-0.27	-	-0.09	-	-	-	
			Driving Lapses				**0.4**	-	-0.56	-	-	-	
			Driving Errors				0.12	-	**0.24**	-	-	-	

			Driving Violations				-0.26	-	-0.63	-	-	-	
2023	The brain network underlying attentional blink predicts symptoms of attention deficit hyperactivity disorder in children	Clinical	ADHD Symptoms - AB	91	Shen 268-node	268	**0.284**	-	0.395	-	-	-	LOOCV
			ADHD Symptoms - TD				**0.326**	-	**0.4**	-	-	-	
2023	The connectome-based prediction of trust propensity in older adults: A resting-state functional magnetic resonance imaging study	Personality	Trust Propensity	120	Dosenbach	142	-	**0.25**	-	0.05	-	-	10-Fold CV
2023	The Individualized Prediction of Neurocognitive Function in People Living With HIV Based on Clinical and Multimodal Connectome Data	Cognitive	Cognitive Function (7 Domains)	102	Power Atlas	264	**0.35**	-	**0.45**	-	**0.54**	-	LOOCV
2023	Trait repetitive negative thinking in depression is associated with functional connectivity in negative thinking state rather than resting state	Clinical	MDD	62	Shen 268-node	230	-	-	-	-	-	**0.826**	5-Fold CV
			Ruminative Response Scale	36 - MDD			-	-	-	-	-	-0.049	
			Ruminative Response Scale	62			-	-	-	-	-	**0.613**	
2023	Using modular connectome-based predictive modeling to reveal brain-behavior relationships of individual differences in working memory	Cognitive	Working memory (0-back d')	874	Shen 268-node	268	-	-	-	-	**0.18**	-	LOOCV
			Working memory (2-back d')				-	-	-	-	**0.18**	-	
2024	Connectome-based prediction of decreased trust propensity in older adults with mild cognitive impairment: A resting-state functional magnetic resonance imaging study	Personality	Trust Propensity	115	Dosenbach	142	**0.41**	-	**0.2**	-	-	-	LOOCV
2024	Connectome-based predictive modeling of Internet addiction symptomatology	Clinical	Internet Addiction	677	Shen 268-node	268	-0.06	-	**0.202**	-	-	-	LOOCV
2024	Connectome-based predictive modelling estimates individual cognitive status in Parkinson's disease	Clinical	Global Cognitive Composite Score	58	368 Shen Atlas	368	-	**0.68**	-	**0.63**	-	**0.59**	LOOCV
2024	Finger motor representation supports the autonomy in arithmetic: neuroimaging evidence from abacus training	Cognitive	Arithmetic Ability	69 - Control	400 ROI Schaefer	400	-	**0.352**	-	0.236	-	-	LOSOCV (subject)
				78 - AMC (received math training prior)			-	**0.391**	-	**0.371**	-	-	
2024	Sex differences in functional connectivity and the predictive role of the connectome-based predictive model in Alzheimer's disease	Clinical	Montreal Cognitive Assessment Scores	71 - Males	AAL	90	**0.23**	-	-	-	-	-	LOOCV
				88 - Females			**0.34**	-	-	-	-	-	

Supplementary Table 2 | Studies selected for literature review of prior studies that use Connectome Predictive Modeling on resting-state fMRI. The 57 papers that passed our exclusion criteria with their respective year, descriptive category, target for prediction, number of subjects, atlas, number of regions of interest, cross validation method, and the model accuracies reported as either Pearson's or Spearman's R. Papers are listed alphabetically, by year. Bolded R values were reported as significant in the original papers. The papers which used Pearson correlation were used to generate Figure 3.d.

*This study reported the CPM accuracy before and after accounting for head motion, age, gender, and intelligence. We report the accuracy after accounting for these confounds, as we account for head motion in our analysis.

Supplementary Table 3.

Year	Title	Exclusion Criteria
2018	Connectome-based models predict separable components of attention in novel individuals	CPM on task data
2018	Connectome-based predictive modeling of attention: Comparing different functional connectivity features and prediction methods across datasets	Review Article
2018	Robust prediction of individual creative ability from brain functional connectivity	CPM on task data
2019	An information network flow approach for measuring functional connectivity and predicting behavior	Substantially modified CPM
2019	Combining multiple connectomes improves predictive modeling of phenotypic measures	Review Article
2019	Connectome-based model predicts individual differences in propensity to trust	Substantially modified CPM
2020	Bootstrapping promotes the RSFC-behavior associations: An application of individual cognitive traits prediction	Incompatible Accuracy Reporting
2020	Functional connectomes linking child-parent relationships with psychological problems in adolescence	Not Whole Brain / ROI Level
2020	Individual variability in functional connectivity architecture of the mouse brain	Non Human data
2021	Context Matters: Situational Stress Impedes Functional Reorganization of Intrinsic Brain Connectivity during Problem-Solving	EEG Study
2021	Dissociable neural substrates of opioid and cocaine use identified via connectome-based modelling	CPM on task data
2021	Impact of binge drinking during college on resting state functional connectivity	Not Whole Brain / ROI Level
2021	NBS-Predict: A prediction-based extension of the network-based statistic	Substantially modified CPM
2021	Neurobiological substrates of the positive formal thought disorder in schizophrenia revealed by seed connectome-based predictive modeling	Not Whole Brain / ROI Level
2021	Optimizing differential identifiability improves connectome predictive modeling of cognitive deficits from functional connectivity in Alzheimer's disease	Substantially modified CPM
2021	Resting-state connectome-based support-vector-machine predictive modeling of internet gaming disorder	Substantially modified CPM
2021	Time-delay structure predicts clinical scores for patients with disorders of consciousness using resting-state fMRI	Substantially modified CPM
2022	Functional connectivity of the central autonomic and default mode networks represent neural correlates and predictors of individual personality	Not Whole Brain / ROI Level
2022	Functional Connectome–Based Predictive Modeling in Autism	Review Article

2022	Individual differences in (dis)honesty are represented in the brain's functional connectivity at rest	Not Whole Brain / ROI Level
2022	Intrinsic functional connectivity in the default mode network predicts mnemonic discrimination: A connectome-based modeling approach	Not Whole Brain / ROI Level
2022	Resting-state functional connectivity of social brain regions predicts motivated dishonesty	Not Whole Brain / ROI Level
2023	A machine learning based approach towards high-dimensional mediation analysis	Substantially modified CPM
2023	Bidirectional connectivity alterations in schizophrenia: a multivariate, machine-learning approach	Substantially modified CPM
2023	Brain networks for temporal adaptation, anticipation, and sensory-motor integration in rhythmic human behavior	CPM on task data
2023	Connectome-based predictive modeling of empathy in adolescents with and without the low-prosocial emotion specifier	Incompatible Accuracy Reporting
2023	Connectome-based predictive modeling: A new approach of predicting individual critical thinking ability	Not Whole Brain / ROI Level
2023	Connectome-based predictive modelling of cognitive reserve using task-based functional connectivity	CPM on task data
2023	Investigating cognitive neuroscience theories of human intelligence: A connectome-based predictive modeling approach	Not Whole Brain / ROI Level
2023	Seeing the future: Connectome strength and network efficiency in visual network predict individual ability of episodic future thinking	Not Whole Brain / ROI Level
2024	Brain-based graph-theoretical predictive modeling to map the trajectory of anhedonia, impulsivity, and hypomania from the human functional connectome	CPM on task data

Supplementary Table 2 | Studies selected for literature review of CPM on resting-state fMRI that were excluded from our comparison, listed with the reason for exclusion. The 31 papers are ordered alphabetically, by year.

Supplementary Figures

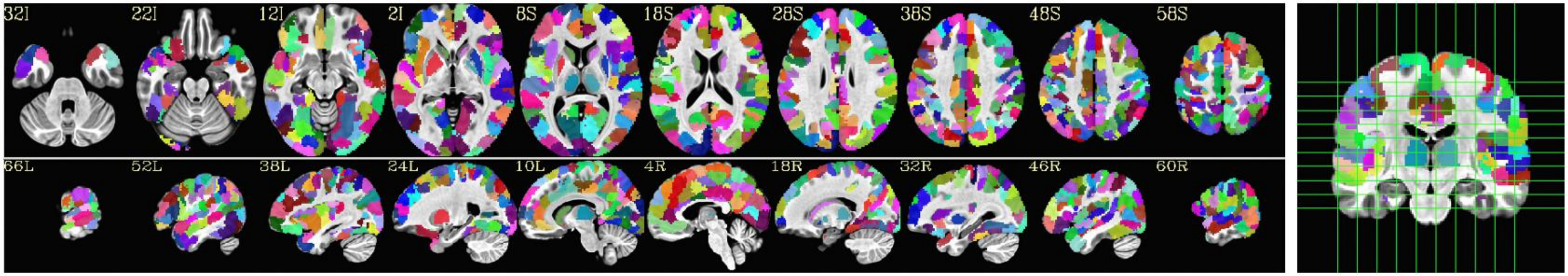

Supplementary Figure 1 | Parcellation used to generate FC matrices. This parcellation is a combination of the 400 ROI Schaeffer Atlas plus eight subcortical regions from the AAL atlas. A few ventral regions with poor coverage in the functional scans were removed. The final number of ROIs in the parcellation is 380 regions.

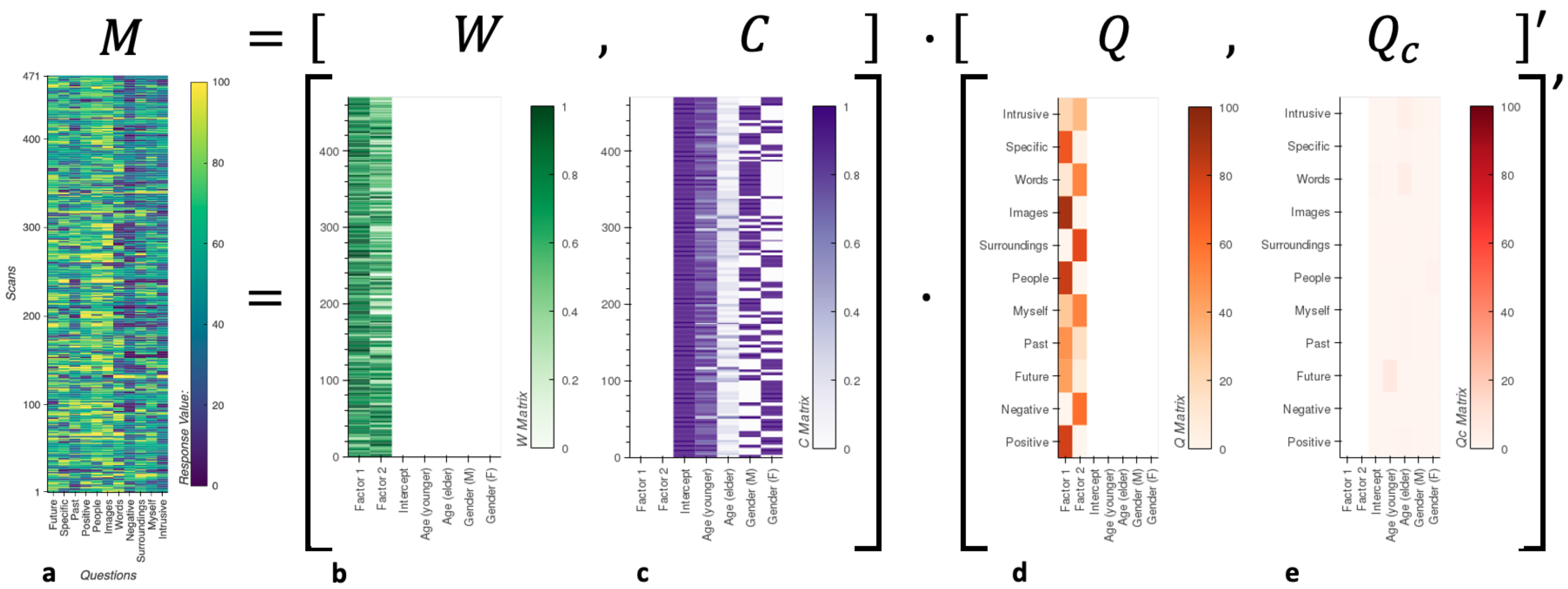

Supplementary Figure 2 | Matrix decomposition of questionnaire items using sparse box-constrained non-negative matrix factorization. **a,** matrix M with answers to all 11 questions for all 471 scans. **b,** Matrix W with 2D representation of the questionnaire data. **c,** Matrix C which encoded demographic information (age range and gender). **d,** Matrix Q, which encodes how each of the two dimensions in W (Factor 1 and Factor 2) relate to the original 11 questionnaire items. **e,** Matrix Qc, which contains information about relationships between demographic information and the way participants answered questionnaire items. All entries in the Qc matrix are small compared to those in the Q matrix, which indicate that demographics does not play an important role as a confound in the data.

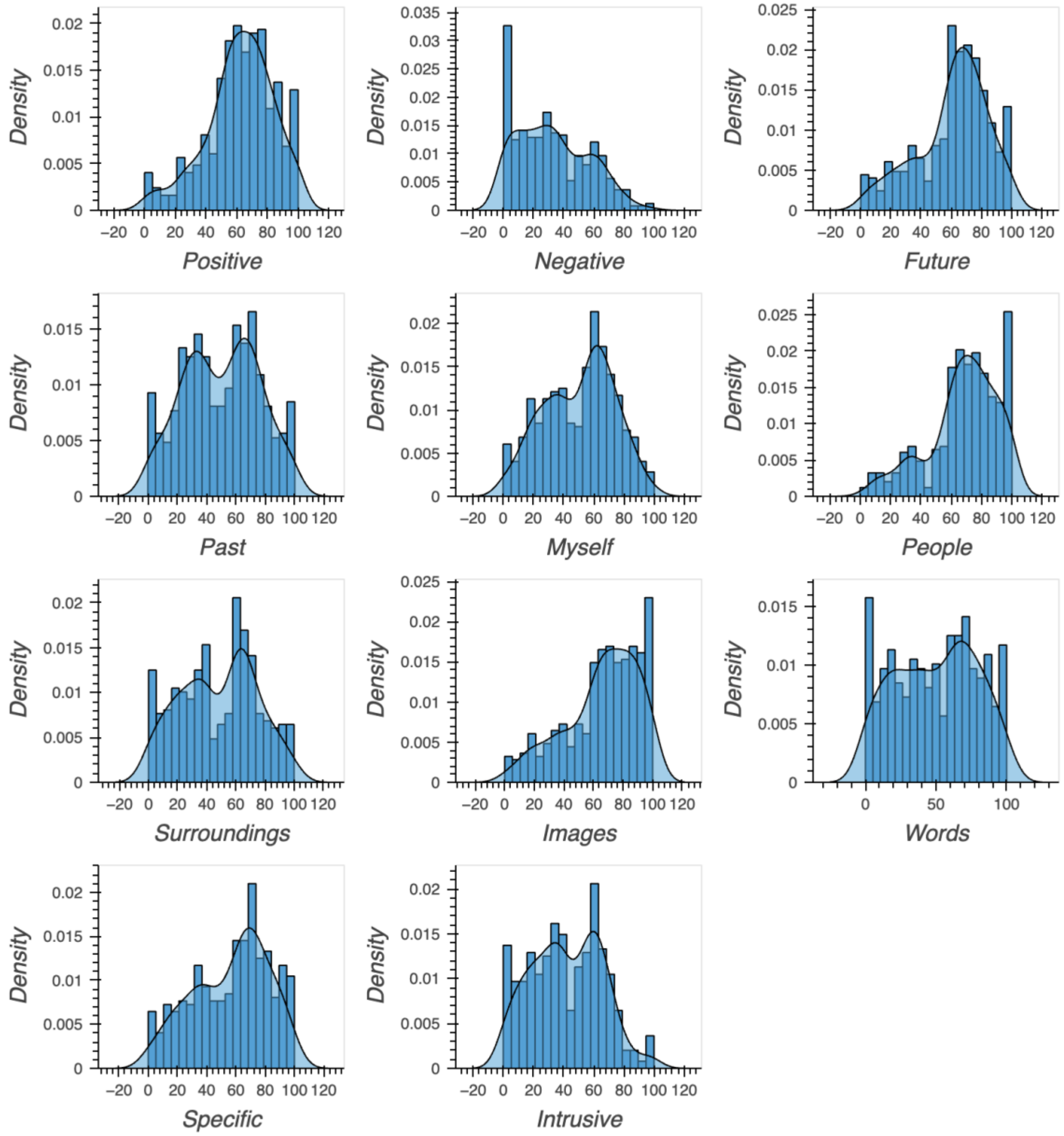

Supplementary Figure 3 | Distribution of answers to the 11 questionnaire items about the form and content of thoughts. Labels on the X-axis match those in Table 1 where we provide the original questionnaire items presented to the participants at the conclusion of each resting-state scan.

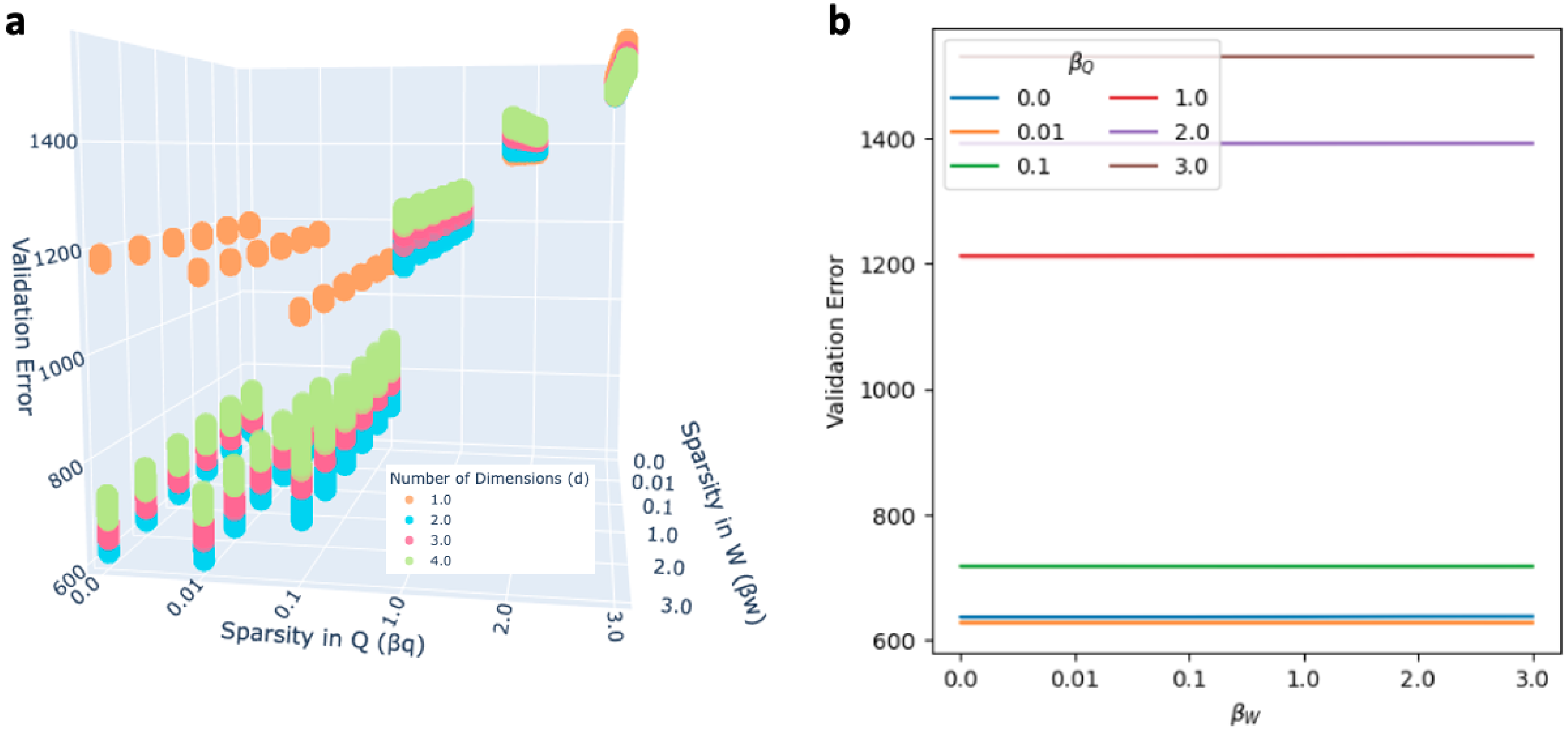

Supplementary Figure 4 | a, Validation error cost function for all explored hyper-parameters. Independently of β_Q and β_W, lowest validator errors are always obtained for *d=2*. **b,** Same validation curve errors, but only for d=2. Here we can observe that the lowest error are obtained for β_Q=0.01 (orange trace). Once β_Q is set that way, there is no difference in error across β_W values. We selected β_W = 0 to avoid unnecessary application of an additional sparsity argument.

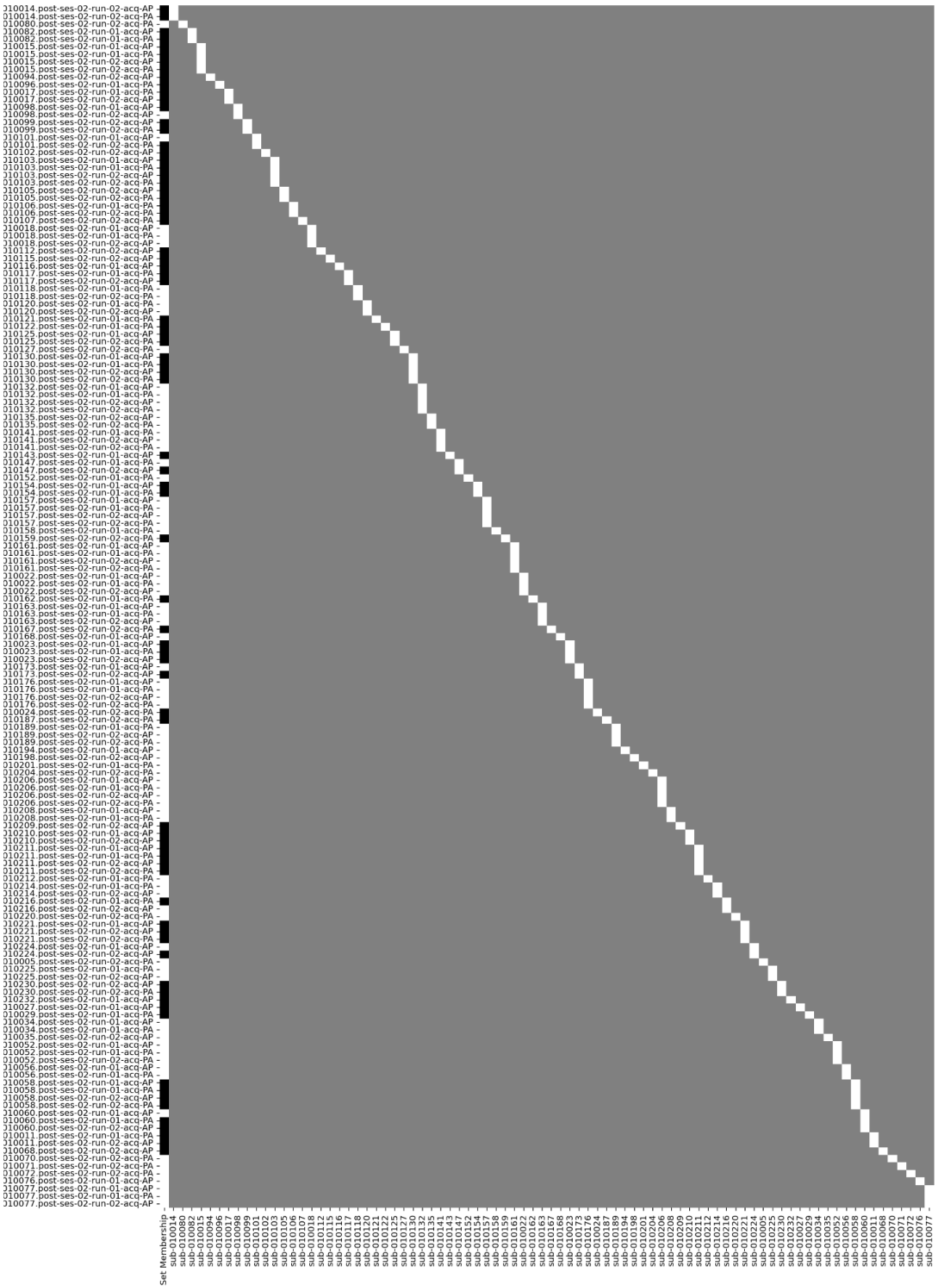

Supplementary Figure 5 | Design Matrix for NBS contrast. Rows corresponds to individual scan. First column contains information about set identity (based on TP1 and TP2). The remaining columns encode subject identity for each individual scan.
